# Participatory development of practical, affordable, insecticide-treated mosquito proofing for a range of housing designs in rural southern Tanzania

**DOI:** 10.1186/s12936-022-04333-0

**Published:** 2022-11-05

**Authors:** Rogath Msoffe, Matilda Hewitt, John P. Masalu, Marcelina Finda, Deogratius R. Kavishe, Fredros O. Okumu, Emmanuel A. Mpolya, Emmanuel W. Kaindoa, Gerry F. Killeen

**Affiliations:** 1 Department of Environmental Health and Ecological Sciences, Ifakara Health Institute, Ifakara, United Republic of Tanzania; 2 Department of Global Health and Biomedical Sciences, Nelson Mandela African Institution of Science and Technology, P. O. Box 447 Arusha, United Republic of Tanzania; 3 Department of Vector Biology, Liverpool School of Tropical Medicine, Pembroke Place, Liverpool, L3 5QA UK; 4 School of Life Science and Bioengineering, The Nelson Mandela African Institution of Science and Technology, P. O. Box 447, Arusha, Tanzania; 5 School of Biological, Earth & Environmental Sciences, Environmental Research Institute, University College Cork, Cork, T23 N73K Republic of Ireland

**Keywords:** *Anopheles*, Malaria, Vector control, House, Mosquito, Ventilation, Netting, Screens

## Abstract

**Background:**

Insecticidal mosquito-proof netting screens could combine the best features of insecticide-treated nets (ITNs) and indoor residual spraying (IRS), the two most important front line vector control interventions in Africa today, and also overcome the most important limitations of these methods. This study engaged members of a rural Tanzanian community in developing and evaluating simple, affordable and scalable procedures for installing readily available screening materials on eave gaps and windows of their own houses, and then treating those screens with a widely used IRS formulation of the organophosphate insecticide pirimiphos-methyl (PM).

**Methods:**

A cohort of 54 households recruited upon consent, following which the structural features and occupant demographics of their houses were surveyed. Indoor mosquito densities were surveyed longitudinally, for approximately 3 months before and over 5 months after participatory house modification and screening using locally available materials. Each house was randomly assigned to one of three study arms: (1) No screens installed until the end of the study (negative control), (2) untreated screens installed, and (3) screened installed and then treated with PM, the insecticidal activity of which was subsequently assessed using standard cone assays.

**Results:**

Almost all (52) recruited households participated until the end, at which point all houses had been successfully screened. In most cases, screening was only installed after making enabling structural modifications that were accepted by the enrolled households. Compared to unscreened houses, houses with either treated or untreated screens both almost entirely excluded *Anopheles arabiensis* (Relative reduction (RR) ≥ 98%, P < < 0.0001), the most abundant local malaria vector. However, screens were far less effective against *Culex quinquefasciatus* (RR ≤ 46%, P < < 0.0001), a non-malaria vector causing considerable biting nuisance, regardless of their treatment status. While PM did not augment household level protection by screens against either mosquito species (P = 0.676 and 0.831, respectively), 8 months after treatment it still caused 73% and 89% mortality among susceptible insectary-reared *Anopheles gambiae* following exposures of 3 and 30 min, respectively.

**Conclusions:**

Participatory approaches to mosquito proofing houses may be acceptable and effective, and installed screens may be suitable targets for residual insecticide treatments.

**Supplementary Information:**

The online version contains supplementary material available at 10.1186/s12936-022-04333-0.

## Background

Across most of sub-Saharan Africa, the most important malaria vector mosquito species are *Anopheles gambiae *sensu stricto (*s.s*.)*, Anopheles coluzzii, Anopheles funestus,* and *Anopheles arabiensis.* The first three species are the most efficient and important vectors but also the most vulnerable to control with long-lasting insecticidal nets (LLIN) and indoor residual spray (IRS) insecticides because they are highly specialized for feeding on humans while they are indoors at night and usually rest indoors too. Even untreated mosquito-proof screening for houses can suppress densities of indoor-feeding mosquitoes, and it is notable that impact appears to vary in proportion to the preference of the mosquito species for human blood [1]. The potential for insecticide-treated house screening may be even greater [1], even for vector species like *An. arabiensis* that are considered behaviourally evasive because they can feed on people or animals outdoors and only visit houses very briefly in search of blood [2].

Historically, the vast majority of malaria transmission in Africa has occurred indoors because mosquitoes were able to freely enter most houses and attack unprotected people [3–6]. Structural features which have been proven to be risk factors for malaria infection include the absence of a celling, open eaves, windows and gaps in the walls, especially around door and window frames, and gaps in the doors themselves [7]. A systematic review and meta-analysis of studies from across Africa indicate that people living in improved housing had, on average, a 47% lower odds of chronically carrying malaria and a 45 to 65% lower odds of experiencing acute symptomatic clinical malaria [8]. Another review article indicates that African children living in modern houses are up to 14% less likely to have malaria compared to the children living in traditional houses [9].

While the epidemiological benefits of house screening have been well documented [7–10], a lack of evidence demonstrating the affordability, acceptability, and practicability of mosquito proofing for typical African houses is still considered an obstacle to scale up [1, 11, 12]. Enabling households to afford and apply a particular house screening intervention in a given context requires careful consideration of what materials are readily available, what community engagement practices may be acceptable to householders and local authorities, and what installation procedures may suit the most basic local house designs occupied by low-income households. Moreover, it is essential that community members and leaders are involved from the outset in development and implementation of approaches for mosquito screening their houses.

Furthermore, to match the full impact that LLINs and IRS have already achieved, largely through mass vector population suppression effects, screened houses will have to do more than just exclude mosquitoes: they will also have to kill them when they attempt to enter or exit the house. Treated netting screens for mosquito proofing houses have several advantages over LLINs and IRS as an insecticide deployment format because it combines essential features of these interventions while also overcoming some of their most important limitations, explained in detail as follows.

Screened housing extends the physical protection of bed nets beyond sleeping spaces, to cover entire domestic spaces and all indoor activities, often resulting in increased usage of those protected spaces [13, 14]. Remarkably, the amount of netting in one ITN may be enough to cover all windows and eave gaps of a typical rural Tanzanian house, so the quantities of netting and insecticide required to protect entire household would be far lower than with several ITNs [15, 16]. House screens are rarely disturbed once installed, so they may last longer than bed nets and more durable netting materials can minimize replacement rates. Like bed nets [17, 18], even untreated window screening can suppress vector populations by denying them access to human blood [1]. Like ITNs, house screening offers a standardized target surface for the durable insecticide treatments required to achieve sufficient impact upon human-specialized African vector populations and the exceptionally intense malaria transmission they mediate [19, 20]. Insecticide-treated house screening could enable affordable deployment of insecticide combinations as mixtures, rotations, mosaics or even micro-mosaics at household level [21], but with far lower application frequency and cost than IRS [15, 22].

The potential of this insecticide deployment format in terms of environmental health are also considerable. First of all, netting screens may be treated by soaking, brushing or rolling, thus eliminating the hazardous aerosols generated by IRS [15, 22]. Second, by reducing recurrent insecticide consumption, application and shedding rates relative to IRS, and reducing physical contact rates relative to ITNs, exposure of both householders and vector control professionals should be reduced [15, 22].

It is therefore possible that insecticidal mosquito screening of houses could supersede ITNs and IRS as front-line interventions of choice for controlling malaria vectors that feed and rest inside human habitations, but only if more practical and affordable formats can be developed [1, 11, 12] that enable scale up towards similar universal coverage targets across entire communities [16, 23, 24]. This study was, therefore, conducted in semi-rural areas at the outskirts of Ifakara town in southern Tanzania, where investigators and householders worked together to: (1) identify and exploit readily available materials around the peri-domestic environment that could be readily used to modify their house structures to make installation of mosquito screening easier and more affordable, and then (2) develop acceptable and practical procedures for modifying the participants houses and then mosquito-proof remaining open eave and window spaces with netting screen materials that were provided free of charge by the study team. Given the importance of lethal insecticides for achieving the full community-wide mass effects of personal protection measures [16, 25, 26], this study also (3) developed a simple procedure of painting an IRS formulation of the organophosphate insecticide pirimiphos-methyl (PM) onto the netting screens that were fitted to the eaves and windows of local houses and, (4) assessed the longevity of these insecticide applications in terms of their efficacy for killing susceptible mosquitoes.

## Methods

### Study area

This study was conducted over a period of ten months, inclusive of household recruitment, in selected neighbourhoods at the periphery of the Ifakara town in south-eastern Tanzania. The neighbourhoods of Katindiuka, Viwanja Sitini, Mlabani, and Lipangala are all found adjacent to extensive malaria vector breeding sites at the edge of the town. Ifakara lies within the Kilombero Valley at − 8.133° latitude, 36.183° longitude and 300 m of elevation. The annual rainfall of the area is 1200–1800 mm, average relative humidity of 63%, and daily mean temperature ranges from 20 to 32.6 °C. Economic activities in the area include crop farming, fishing and brick-making among others.

Historically, Ifakara town has always had far lower rates of malaria transmission than the surrounding valley [27–29]. Since the scale-up of insecticide-treated nets, expanding urbanization, improved housing and living standards, malaria transmission has further declined and the formerly-dominant vector, *An. gambiae* has essentially disappeared [30, 31]. Recent surveys in the villages just south of Ifakara have estimated residual EIRs of approximately 4 infectious bites per person per year for *An. arabiensis* and 12 infectious bites per person per year for *An. funestus* [32]. Both of these two most important vector species in the Kilombero Valley area are now resistant to the pyrethroid, carbamate and organochlorine insecticides used for vector control in LLIN and IRS formats [33, 34]. Communities living in the valley have high levels of access to bed nets and use them extensively, some of which are pyrethroid-treated and some of which are not, because they are distributed through several mechanisms, either free of charge from the government or routine sales from retailers [35]. There is currently no IRS treatment that is systematically applied to houses in this area.

### Characteristics of houses found in the study site

Most houses in and around Ifakara have walls constructed of baked mud bricks, held together with either mud or concrete [36], and most roofs are made of corrugated iron sheets [37]. The structure, size and design of houses in this area are highly variable but some features that allow mosquitoes to enter are very common and represent suitable targets for improved screening procedures. Such features include the eave gaps left open between the roof and wall, open window spaces and gaps around doorframes, cracks in walls and other gaps in their structures, many of which are designed to facilitate natural ventilation. In this study, houses that had exactly these open structural features and also lacked netting screens to block mosquito entry were selectively enrolled.

### Overall study design and sample size calculations

This study consisted of observational and experimental components. First of all, direct observations and informal discussions were used to assess the availability of materials and acceptability of various potential structural modifications for mosquito proofing houses. Then brief surveys recorded the demographic characteristics of the participating households and the structural characteristics of their houses.

The experimental component of the study consisted of a controlled evaluation of two mosquito-proofing intervention options through randomized assignment of participating households to one of three intervention arms: (1) No screens on eaves and windows (Negative control), (2) screens installed on open eaves, windows and other mosquito entry points and (3) screens installed and then treated with PM insecticide. The primary outcomes were indoor densities of *An. arabiensis* and *Culex quinquefasciatus* mosquitoes in each of the intervention arms, measured approximately monthly for about three months before and over five months after participatory house modification and screening. Secondary outcomes were the proportional mortality rates of fully susceptible insectary-reared *An. gambiae* mosquitoes, 72 h after exposures to netting screen for either 3 or 30 min, measured 4 months and 8 months after treatment.

Sample size calculations for determining the number of houses required were based on the primary quantitative outcome, which was defined as the number of *An. arabiensis* mosquitoes caught per house per night of trapping. In order to leave a substantial margin for error, a worst-case-scenario was assumed with very low vector densities of only four specimens per unscreened negative control house per night of light trap capture, approximately representative of a typical dry season conditions in this setting. Applying Lehr’s equation for calculating sample sizes for Poisson-distributed count outcomes [38], it was estimated as follows that 12 houses would be required per study arm to detect a 50% reduction of indoor biting density with a power of 80% and probability threshold of 5%. A total of 54 houses were therefore recruited to allow for drop out and also to ensure this minimum of 12 houses was achieved, especially despite the fact that the intervention allocation procedure involved randomization with replacement, resulting in some arms having lower numbers of houses than others through simple chance.

### Community engagement, house selection and household recruitment

Relevant village council leaders were sensitized to the goal, objectives and procedures of the study through the regular quarterly IHI community engagement meetings conducted in *kiswahili*. The investigators then selectively engaged with Ten-Cell Unit (TCU) housing clusters (*mashina*) in the study site neighbourhoods (*mtaa*) nearest to the periphery of Ifakara town, with priority given to those whose elected TCU leaders (*mabalozi/wajumbe*) and neighbourhood chairpersons (*wenyeviti*) expressed greatest enthusiasm for facilitating the study. In these selected TCUs*,* community sensitization was conducted in situ at their peri-domestic household environment with the help of these TCU leaders and neighbourhood chairpersons.

Participants in this study were the members of purposively selected households, recruited through their adult (18 years or older) household heads. Specifically, this study aimed to recruit households with unscreened houses who could benefit most from participation. Interestingly, now that Ifakara has been progressively urbanized over the last two decades, such unscreened houses proved to be relatively rare even at the outskirts of the town, so no interested households had to be declined an opportunity to participate. Informed consent to participate from the respective household heads was documented in writing following detailed explanation of the projects aims, procedures, potential risks and potential benefits. The inclusion criteria were houses that had less than or equal to six rooms in one building, lacked screening on windows, eave gaps or ceilings, in which most occupants are long-term residents who all live within the study area. Consenting participants were subsequently allowed to withdraw their houses and households from the study at any time. Where it was possible to ask the household to explain their reasons for withdrawing, the researchers documented their self-reported rationale.

### Allocation to intervention treatment arm by participatory randomization with replacement

**Fig. 1:**
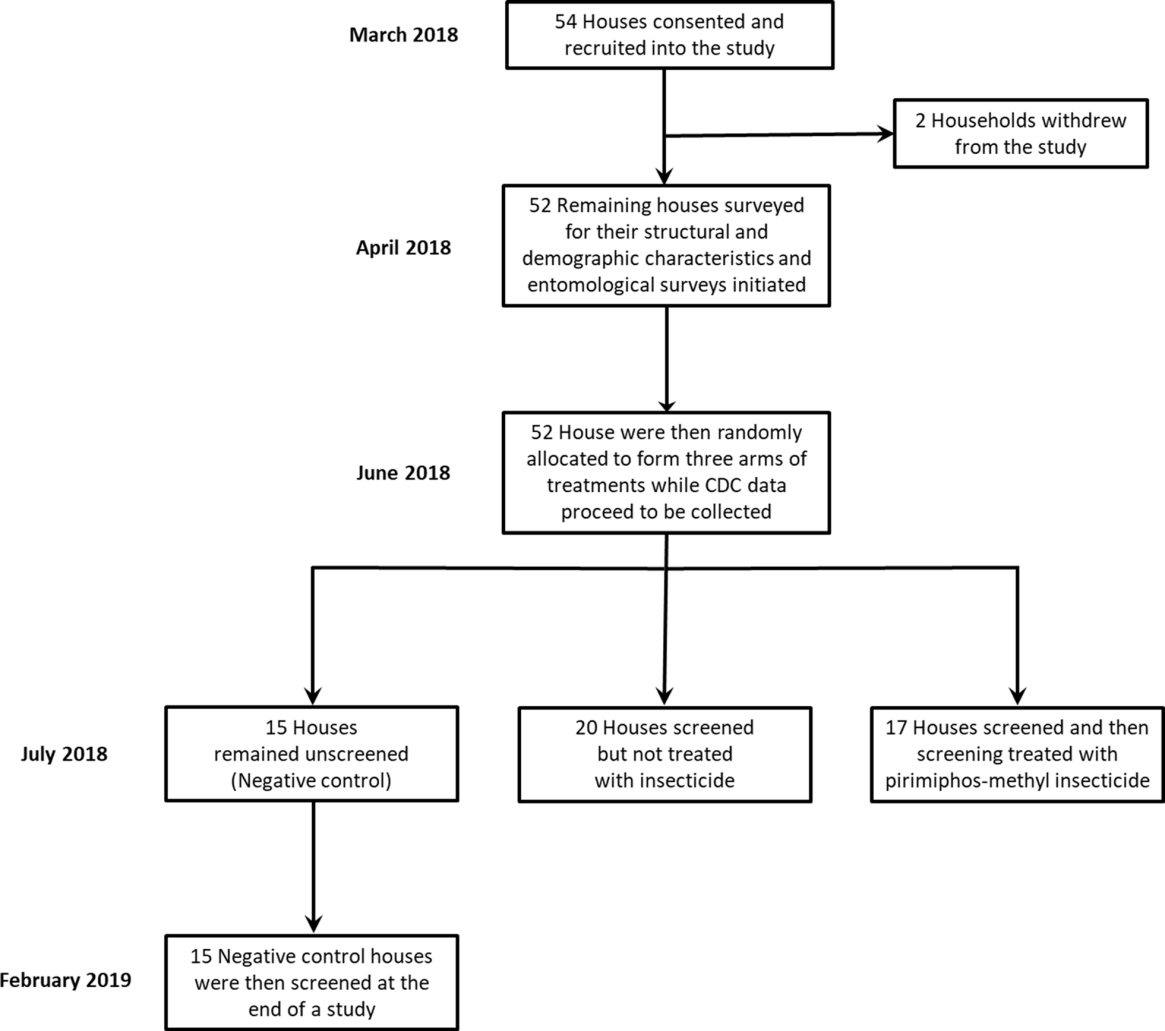
Flow diagram illustrating the study design, as well as the recruitment, retention and treatment allocation outcomes for all consenting households who participated in the study

Each participating household head picked only one of three different-coloured marbles from an opaque paper bag, with each of the three colours chosen a priori to represent one of the three treatment arms. After each householder had picked his or her marble at random and understood what intervention treatment his or her house would receive, that marble was returned to the bag and remixed with the others before the next household head could make his or her selection. In this way, each household had a transparent and equal opportunity of being allocated to whatever they consider the most desirable treatment arm, regardless of the allocations of previous participants. The overall outcome of the recruitment, randomization, retention and follow-up procedures are summarized in Fig. 1.

Monthly entomological surveys of all participating houses, as described in detail below, were continued until the end of the study and any relevant changes to the structural characteristics of the houses (including modifications and screening installations to exclude mosquitoes) were recorded as they occurred.

### Participatory development of scalable procedures for mosquito-proofing houses

Researchers spent much of their time within the peri-domestic environment surrounding the houses of the study participants, discussing potential procedural options for affordable, practical, and acceptable procedures for mosquito-proofing their houses, during which time the investigators also learned a lot opportunistically through informal participant observations. All such conversations were conducted with individual household members and no group discussions were conducted, so that each participant could freely share his or her own perspectives, and represent those of other family members, without any peer influence from other participating households. Such direct but informal discussions with the study participants in and around their own houses, made the discussion more practically effective, because household features like as structure, size, dimension and materials, as well as design of house modifications and the screening installations themselves, could be seen, touched and explored.

### Evaluating the impact of mosquito proofing with and without insecticide treatments upon indoor mosquito densities

The impact of the new mosquito-proofing procedures upon indoor mosquito densities was assessed by measuring the number of mosquitoes caught in each house approximately once per month, although sometimes this varied in between houses and periods for practical reasons, such as households being away or busy when visited as per schedule. Indoor host-seeking mosquitoes were collected using CDC light traps hung overnight beside an occupied bed net inside a participating house [39]. Captured mosquitoes were then killed by freezing, identified using morphological keys [40] and sorted by sex and abdominal status. For each room where the trap was hung, if any of the occupant(s) lacked bed net at that time, a new untreated bed net (Safi net®) was provided free of charge to both protect the occupant(s) and enable the effective function of the trap [18, 41].

### In situ treatment of installed netting screens with IRS insecticide formulations

A soft-paint brush of 2 inch width was used to apply an aqueous suspension of micro-capsulated PM (Actellic® 300CS, Syngenta AG, Switzerland) onto mosquito-proof nettings screens after they had been installed. Following saturation calibration experiments to estimate how much aqueous suspension the PVC-coated fibreglass netting used to screen houses could hold (100 ml per m^2^), the working concentration of PM required to achieve a treatment dosage rate of 1 g per metre squared of netting was calculated as 10 g per l, prepared by diluting 33.3 ml of stock concentrate into each litre of water. Application was conducted using personal protective equipment (rubber gloves, face mask, plastic apron and rubber boots) to protect the operator. To prevent environmental contamination, plastic trays were used to collect falling insecticide droplets and plastic bottles were used for temporarily storing unused insecticide suspension. All such aqueous waste, including the triple washes of all plastic containers that were used to clean them, were disposed of in a charcoal-lined soak pit as per existing approved IRS protocols for Tanzania [42]. After they had been triple rinsed with water, all such containers were disposed of as routine plastic waste as per standard protocol [42].

### Assessments of persisting insecticidal activity of treated netting screens

The insecticidal activity of PM-treated netting screens installed on the eaves and windows of participating houses was measured as an indicator of potential for reducing population-level survival and density if scaled-up across entire communities. This enabled determination of proportions of insectary-reared mosquitoes that died following controlled exposures to treated or untreated netting using the standard World Health Organization (WHO) cone bioassay tests [43].

### Statistical analysis

All statistical analyses were done using R version 4.1.0 open-source statistical software, supplemented with the *ggplot2*, *cluster*, *factoextra* and *lme4* packages. First, the structural and household demographic characteristics of enrolled houses were visually examined by plotting them out as density distribution plots with the *ggplot2* package and then summarized in terms of their medians and ranges, as well as their first and second quartiles. Then a partition around medoids cluster analysis was applied, using the *cluster* and *factoextra*, packages to identify two subsets of houses with distinct structural characteristics, for which stratified graphic and tabular summaries were prepared as described immediately above. Then, Poisson regression was used to assess the effect of house screening treatment upon indoor mosquito catches, the primary outcome of this study.

This was accomplished using Generalized Linear Mixed Models (GLMMs) fitted with the *lme4* package, using a Poisson-distribution for the dependent variable (total counts of female mosquitoes from a given taxon per light trap catch), treatment arm allocation and current intervention status as fixed categorical independent variables (1: Unscreened, 2: Screened but not treated, or 3: Screened and treated, with unscreened being the a priori default reference group in both cases, although screened but untreated was treated as the reference group for post hoc comparisons with the treated screens), accounting for the effects of location (house identity) and time (date) by including them as random effects. Overdispersion was accounted for by treating each observation (trap catch in a particular house on a particular night) as an additional random effect.

To examine the influence of the housing structure categories identified by cluster analysis upon the effects of screens on mosquito densities, statistical power was enhanced by combining all screened houses into a single category, so that the contrast made was screened houses, regardless of the insecticide treatment status of those screens (which was first confirmed to have no apparent effect), versus unscreened houses. Also, structural category (well-ventilated versus poorly ventilated) was included as a second independent variable and the interaction between these two (screened and well-ventilated versus all other possible combinations) was included as the third categorical independent variable.

The efficacy of insecticide-treated screens at killing mosquitoes was also assessed using similar GLMMs fitted with *lme4* but with a logit link function and binomial distribution for the dependent variable (proportion of mosquitoes which died), with survey round (4 versus 8 months after treatment), dust cleaning status (before or after wiping) and netting screen treatment status (treated versus untreated screens) as the independent variables and with replicate, house and date as the random effects.

## Results

### Structural and demographic characteristics of the enrolled houses.

**Fig. 2:**
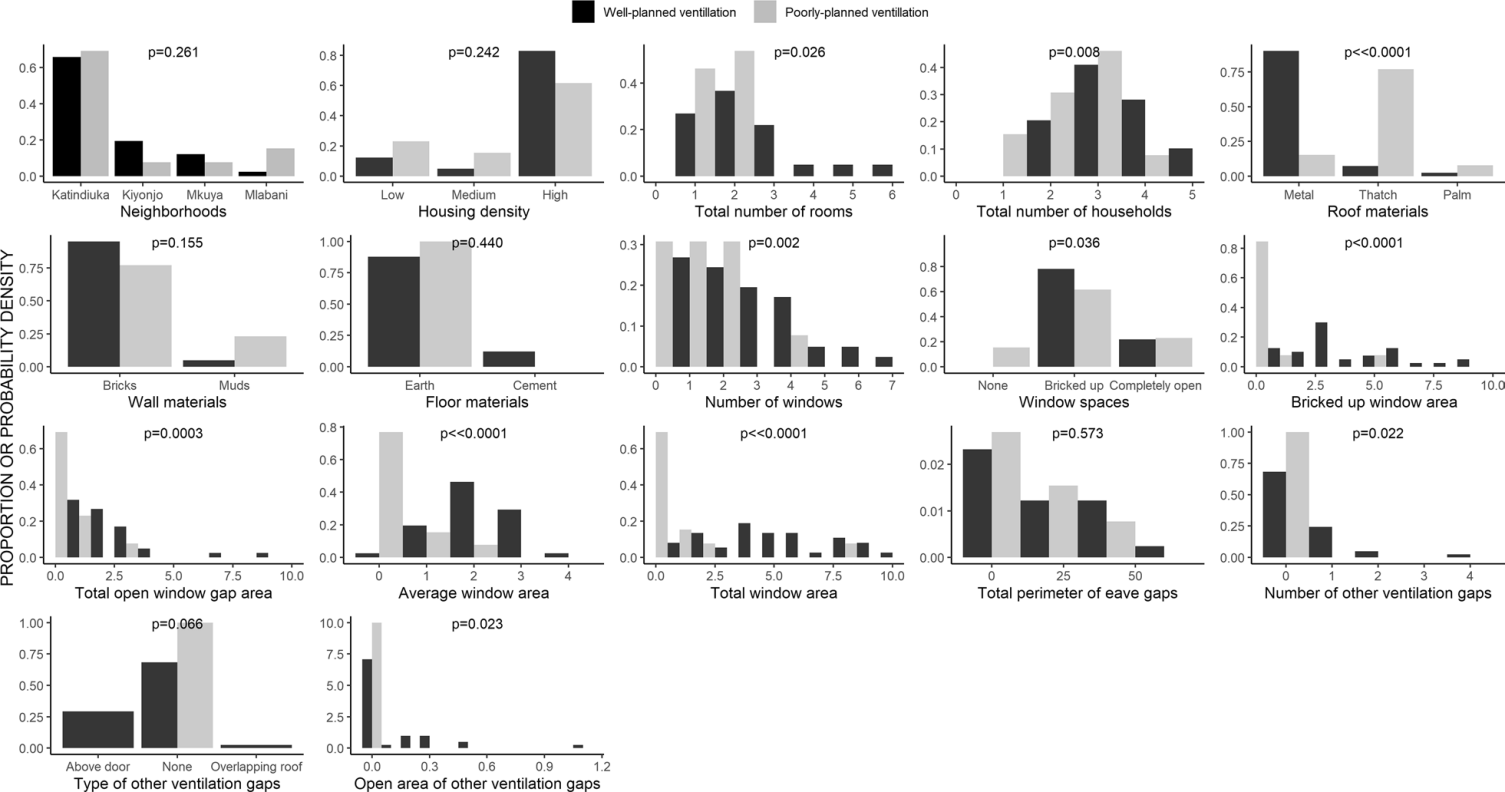
Physical characteristics of houses enrolled in the study, stratified into the two distinct clusters of poorly and well-ventilated houses identified through partition cluster analysis (Fig. 3 and methods). See Table 1 for numerically explicit representation of the same data

**Fig. 3:**
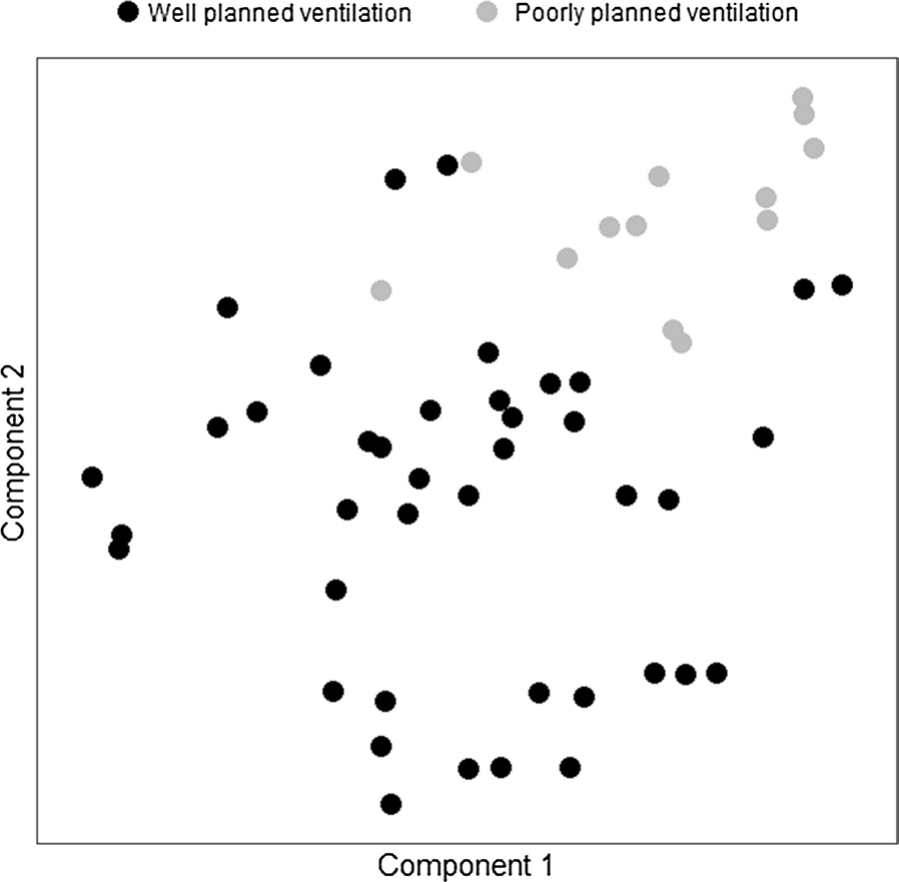
A partition clustering plot illustrating how Partition Around Medoids analysis distinguishes two clusters of houses with different characteristics (Table 1), represented across two dimensions obtained by dimensionality reduction with Principal Component Analysis

The physical characteristics of the study houses are summarised in Table 1. All the recruited houses could be classified as belonging to one of two major clusters, each of which contains houses with similar traits that clearly differ from those in the other cluster (Table 1, Fig. 2). With a small minority of exceptions, most of the houses recruited into the study could be unambiguously categorized into one of two groups, the characteristics of which are compared and contrasted in Table 1 and Figs. 2 and 3: (1) Traditional houses built with poorly planned ventilation and (2) improved houses of more contemporary design with well-planned ventilation.

**Table 1:** Physical characteristics of houses enrolled in the study and statistical contrasts between the two distinct clusters of housing identified through partition cluster analysis (Fig. 3)

Attribute	All houses n [Proportion] for discrete attributes or *Median [Range]* for continuous attributes	Cluster 1 houses (Poorly ventilated) n [Proportion] for discrete attributes or *Median [Range]* for continuous attributes	Cluster 2 houses (Well ventilated) n [Proportion] for discrete attributes or *Median [Range]* for continuous attributes	Statistical contrasts between clusters Χ^2^ test for discrete attributes or Wilcoxon rank sum test for continuous attributes
*Neighborhoods*				
Katindiuka Kiyonjo Mkuya Mlabani	36 [66%] 9 [17%] 6 [11% 3 [6%]	9 [69%] 1 [8%] 1 [8%] 2 [15%]	27 [66%] 8 [20%] 5 [12%] 1 [2%]	Χ^2^ = 4.0, P = 0.261
*Roof material*				
Metal Thatch Palm	39 [72%] 13 [24%] 2 [4%]	2 [15%] 10 [77%] 1 [8%]	37 [90%] 3 [7%] 1 [3%]	Χ^2^ = 28.3, P < 0.0001
*Wall material*				
Bricks Mud and wattle	49 [91%] 5 [9%]	10 [77%] 3 [23%]	39 [95%] 2 [5%]	Χ^2^ = 2.0, P = 0.155
*Surrounding housing density*				
Low Medium High	8 [15%] 4 [7%] 42 [78%]	3 [23%] 2 [15%] 8 [62%]	5 [12%] 2 [5%] 34 [83%]	Χ^2^ = 2.8, P = 0.242
*Ground floor*				
Earth Cement	49 [91%] 5 [9%]	13 [100%] 0 [0%]	36 [88%] 5 [12%]	Χ^2^ = 0.6, P = 0.440
*Window space*				
None Partially/completely bricked up Completely open	2 [4%] 40 [74%] 12 [22%]	2 [15%] 8 [62%] 3 [23%]	0 [0%] 32 [78%] 9 [22%]	Χ^2^ = 6.7, P = 0.036
*Other ventilation gap notes*				
Above a door Between overlapping roofs None	12 [22%] 1 [2%] 41 [76%]	0 [0%] 0 [0%] 13 [100%]	12 [29%] 1 [3%] 28 [68%]	Χ^2^ = 5.4, P = 0.067
Number of rooms	2 [1,6]	2 [1,2]	2 [1,6]	W = 371, P = 0.026
Number of resident households	3 [1,7]	3 [2,7]	3 [1,4]	W = 392, P = 0.008
Eave gap perimeter (m)	12 [0,64]	8 [0,45]	13 [0,64]	W = 294, P = 0.573
Number of window spaces	2 [1,6]	1 [0,4]	2 [1,7]	W = 415, P = 0.002
Window space average area	1.9 [0,3.5]	0.3 [0,2.0]	2 [0.2,3.5]	W = 501, P < 0.0001
Window space total area	3.8 [0,20.2]	0.4 [0,81]	5.3 [0.5,20.2]	W = 488, P < 0.0001
Window space area bricked up	2.6 [0,16.5]	0 [0,4.8]	2.7 [0,16.5]	W = 474, P < 0.0001
Open window gap total area	1.1 [0,8.7]	0.2 [0,3.2]	1.7 [0,8.7]	W = 444, P = 0.0003
Number of other ventilation gaps	0 [0,4]	0 [0,0]	0 [0,4]	W = 351, P = 0.022
Open area of other ventilation gaps	0 [0,1.1]	0 [0,0]	1 [1]	W = 351, P = 0.023

The former consisted entirely of traditional small houses, with only open eaves and small window spaces, if any, as the sole means of ventilation, and walls made with a variety of materials, ranging from baked or unbaked mud bricks to, in a few cases, mud and wattle. With some exceptions, described immediately below, the latter category consisted mostly of larger, taller houses of more contemporary design, built with larger windows spaces and higher ceilings, plus additional ventilation sites that were clear deliberately planned and built into the upper walls, immediately above the doors or between two overlapping roofs. While some of these houses designed with improved ventilation in mind had open eaves, the majority had closed eaves. A minority of houses in this latter category, however, were recently built smaller houses consisting of only one room, but which were taller than traditional, poorly-ventilated houses and had larger eave and window spaces, although the latter were usually fully or partially bricked up. For brevity, houses belonging to the former category are referred to as *poorly ventilated* throughout the remainder of this article, while the latter are described as *well-ventilated*. In contrast with the wide variation in building materials used for the walls and roofs of the poorly ventilated houses, those in the well-ventilated category almost all had walls made of baked bricks and almost all had roofs made of iron sheets.

**Fig. 4:**
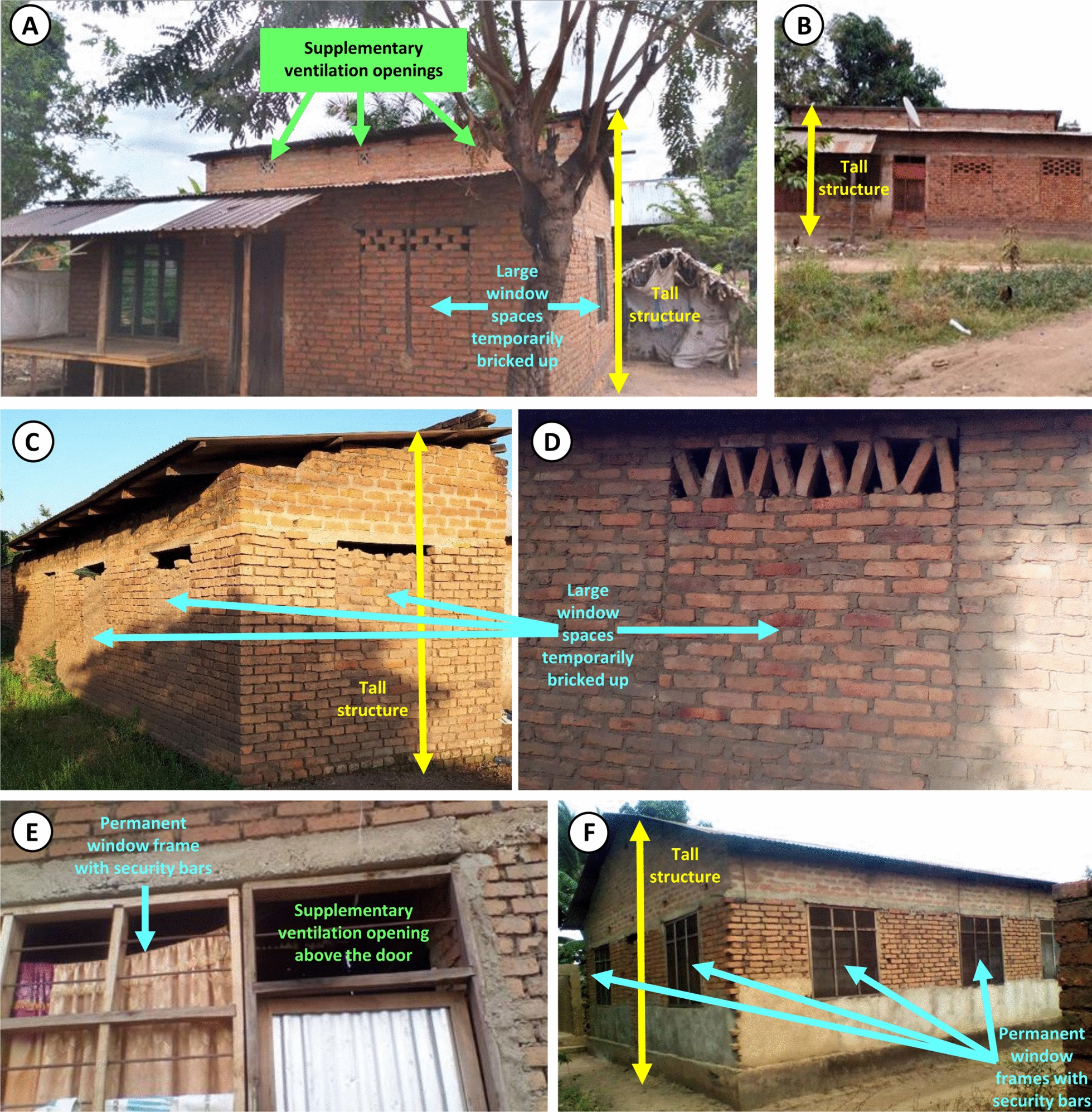
Typical examples of structural features of houses from cluster 2, typically of contemporary design with better a priori ventilation planning, and usually built with brick walls and iron sheet roofs, representative of various stages of completion. **A**, **B** Illustrate how a house may be built in two halves, with the second being an annex to the first, necessitating construction of a small upper wall between the overlapping roofs where additional openings for ventilation and lighting may be built into the structure (**A**). **C**, **D** Illustrate typical examples of temporary brickwork structures used to fill in deliberately constructed window gaps, usually for several years while the household saves enough money to install window frames and security bars (**E**, **F**). Note that the house depicted in **D** was the one identified by the cluster analysis as the *medoid* of the second cluster of houses with well-planned ventilation features*,* meaning it was the most statistical representative member of that cluster of houses

Householders explained that the window spaces of so many houses were bricked up (Table 1) because most houses in the area are built in a stepwise manner over several years, through repeated bouts of construction whenever household cashflow and time availability allowed. For example, the two houses depicted in Fig. 4A and B were both built in two halves, with the second half being built out as an extension of the first, thus resulting in the two corresponding roofs being separated by a small section of wall between the two where they overlap. It is therefore common practice for the window openings to be bricked up for security reasons (Fig. 4A–D) for several years, until the household can afford to install permanent conventional window frames and screens (Fig. 4E, F).

Note, however, that these window spaces are usually only partially bricked up, leaving either completely open spaces (Fig. 4C, D) or partially open brick lattices (Fig. 4A, B) at the top, to allow airflow and light into the house. It was also noted that the eaves of such houses with temporarily bricked up window spaces are often necessarily left open for ventilation and lighting. Some participants explained that the eventual installation of permanent window frames (Fig. 4E, F) is usually accompanied by closing of the eave gaps (Fig. 4A, B, D and F) because the latter then become redundant as a means of ventilation and continue to allow mosquitoes to enter despite the windows themselves being fully screened (Fig. 4E, F).

**Fig. 5:**
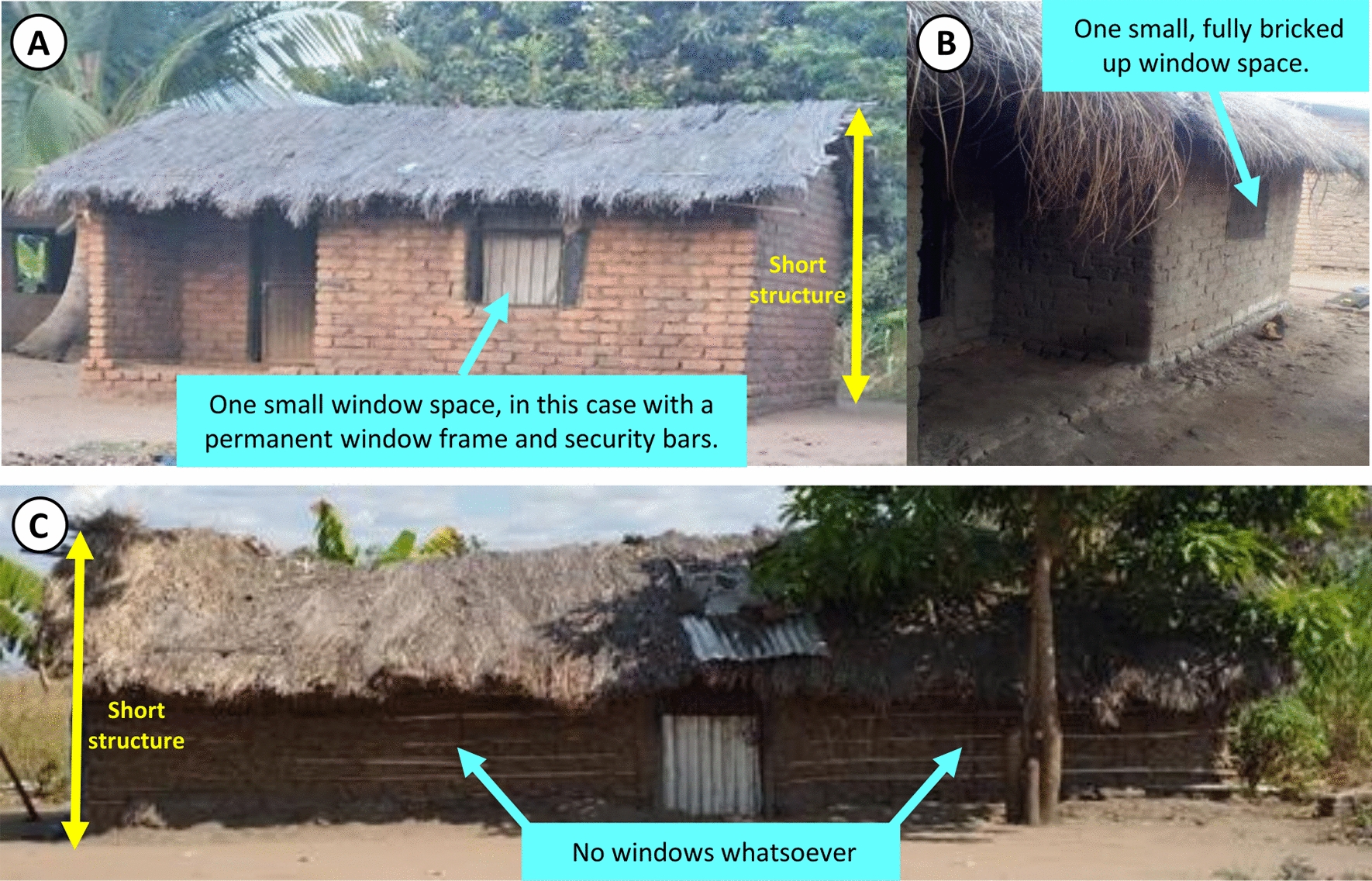
Representative examples of the cluster one houses with poorly planned ventilation based on traditional designs, which were often built with traditional materials like mud and wattles or unbaked bricks for the walls and grass thatch or palm leaves supported by bamboo poles for the roofs

In contrast, the first cluster was mostly comprised of small houses that were not designed so deliberately to maximize airflow and light entry, and usually had fewer rooms, narrower eave gaps and smaller window spaces, if any (Fig. 5). Although some of them had brick walls, others were made of mud and wattle and most were roofed by locally available natural materials, such as thatch/palm leaves. All were occupied by low-income householders and, unlike the better ventilated houses, none had a cement floor.

Through observation and informal discussion with study participants, the investigators came to understand that all of the poorly ventilated houses with bricked up window spaces and (Fig. 5A, B), were constructed a long time ago by elders who transitioned traditional designs using mud and wattle (Fig. 5C) to baked (Fig. 5A) or unbaked (Fig. 5B) bricks without changing the overall structural plan. Consistent with the observations of recent studies in the area [44] it was found that only a small proportion of mud-and-wattle houses remain in the semi-urban setting of Ifakara town, comprising only 6% (3/54) of the enrolled houses despite the fact that only houses with open structures and incomplete screening against mosquitoes were selected for inclusion in this study. All three of these mud and wattle houses were essentially temporary structures with no windows, open eaves and thatch roofs (Fig. 5C). This small number of mud, wattle and thatch houses were observed to be predominantly small, peripherally located and seasonally occupied until the occupants can afford to build a permanent brick-walled house. Seasonal livelihoods practiced by these households included farming and fishing, and temporary occupancy was sometimes motivated by funerals and other occasional ceremonies.

### Practical procedures for screening installation

**Fig. 6:**
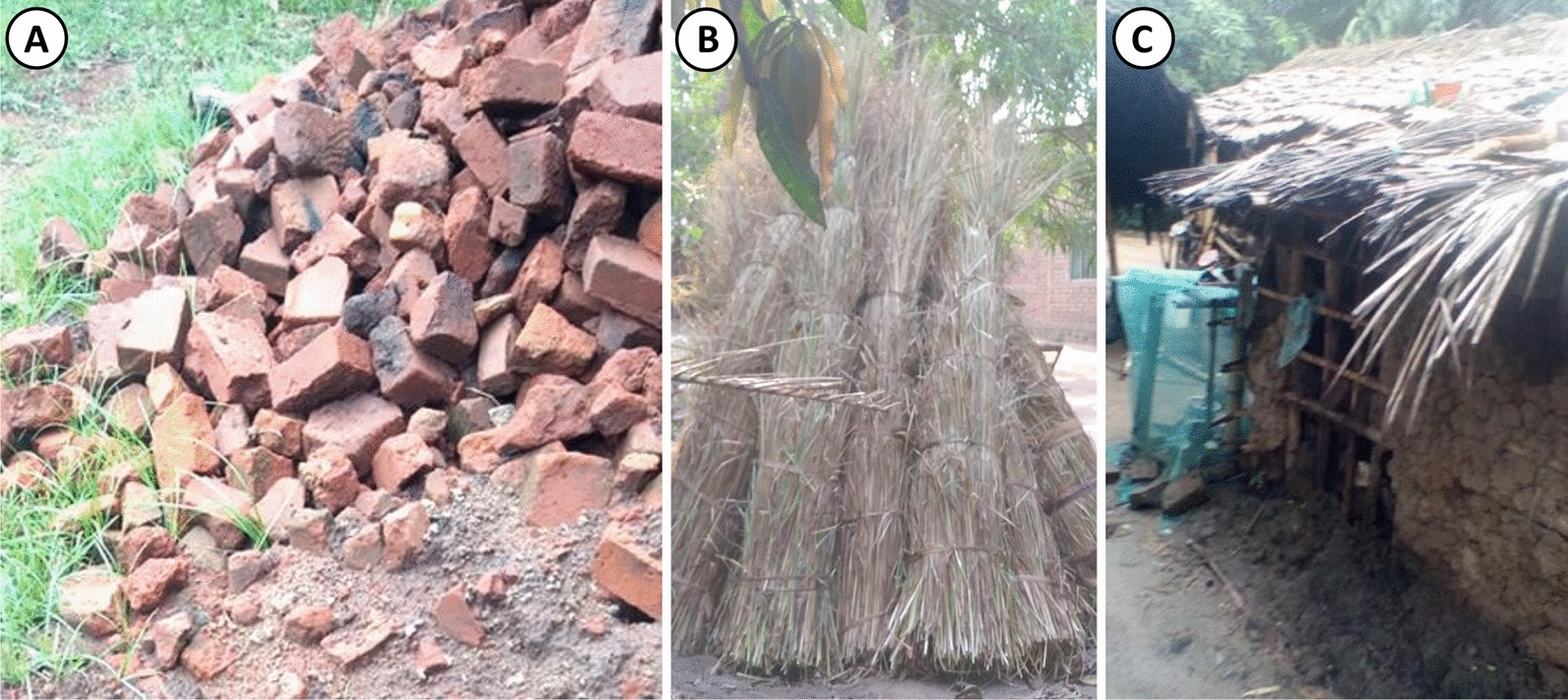
Examples of readily available traditional construction materials in and around environment of the recruited houses. **A** Remaining bricks and brick rubble left over after construction of a house. **B** Bundles of grass thatching collected nearby, which are commonly used for roofing. **C** Palm leaves used to roof a small mud and wattle house

**Fig. 7:**
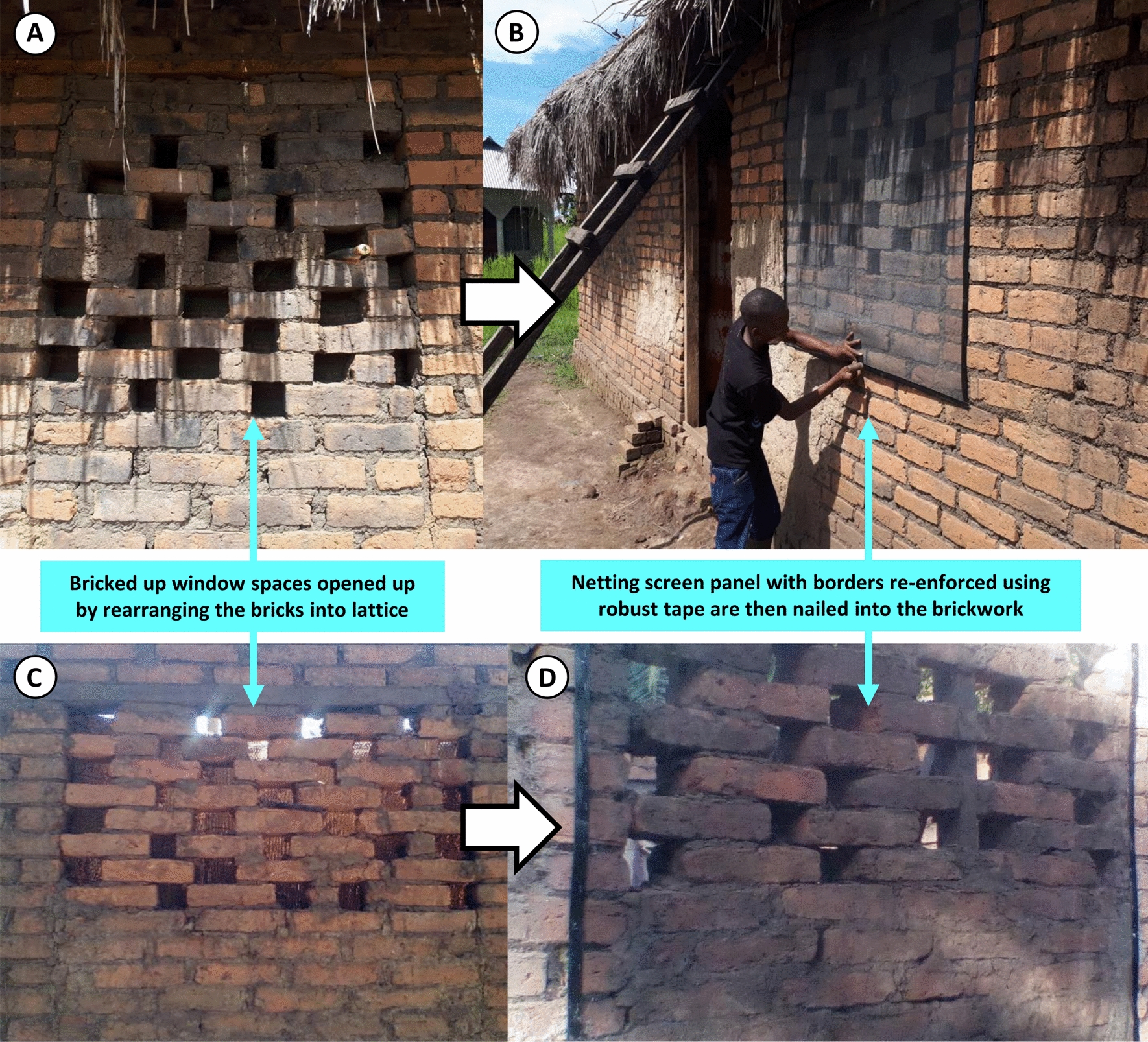
Examples of brickwork lattices and low-cost netting installations used as an interim solution for mosquito-proofing window gaps in brick houses, while also allowing improved lighting and ventilation, for households that could not yet afford to install permanent window frames with security bars (Figs. 4E, 3F). The lattices were formed with bricks and brick rubble, usually just by rearranging the materials that had already been used to fill in the window space completely or nearly completely. The netting installation consisted simply of standard PVC-coated fiberglass screening cut into appropriately sized panels, fitted with robust borders by folding strips of heavy-duty adhesive tape along their edges, which was then reinforced with staples before then nailing into position, directly into the brickwork surrounding the window gap

Materials for mosquito-proofing (Fig. 6) were acquired almost exclusively through one of two major ways: (1) Traditional construction materials that were readily available at no cost in and around the house itself (Mud bricks and brick rubble, mud, tree branches, bamboo, grass and palm leaves), which were used to modify the overall structure of the house to make it much easier to fit netting screens to (Fig. 7), and (2) Affordable hardware readily available for purchase through the retail sector in Ifakara town (PVC-coated fiberglass mosquito screening staples, nails, and string), all of which were procured and installed by the project team free of any charge to the household. The only item that had to be procured from outside of Tanzania, rather than from local vendors in Ifakara, was an unusually robust brand of adhesive tape (Gorilla Tape®) that proved appreciably superior to equivalent products available in Ifakara.

Figure 5A shows the design, shape and appearance of a house built with brick walls and a thatched roof with only one small window for light and ventilation, similar to the most representative medoid house for the second cluster of poorly ventilated houses, which had been demolished and could no longer be photographed by the time of writing. On other hand, Fig. 5B shows a typical small house with walls made of unbaked mud bricks house with a veranda used as a sheltered seating and storage area. The large mud and wattle house depicted in Fig. 5C had no windows, was short in height but occupied a quite long footprint to accommodate the two bedrooms that were separated by a central kitchen room for keeping house utensils and cooking during the rainy season because this house lacked a veranda. It was notable that this house was often heavily contaminated with dust, ashes and smoke because of this indoor kitchen.

Based on informal discussions with some of the participants, it was concluded that it was essential to avoid compromising their pre-existing willingness-to-pay for conventional, permanent window installation formats that include full frames, security bars and grills (Fig. 4E, F). These participants confirmed that it was their intention to follow that same normal practice over the long term and agreed that the modifications the investigators could help them make were intended as a medium term “stopgap” solution for a few years. The study team therefore agreed to work with them to develop the following broadly applicable formats, which were affordable enough to have potential for population-wide scale up and were viewed by the households as an acceptable interim solution until they could afford to install permanent, fully-secure mosquito-proofed window frames (Fig. 4E, F) themselves and then close off remaining access points for mosquitoes, particularly the eaves, on a long-term basis.

From a practical perspective, houses required two distinct approaches to participatory netting screen installation, both of which occurred in both of the identified housing clusters: (1) Houses designed a priori with window spaces, even though most of these were at least partially bricked up on a medium-term basis. For such houses, it was usually possible to close the eaves with consent of the household soon after they had already consented to open up the brickwork used to fill the window space into lattices and had a chance to experience the correspondingly improved lighting and airflow. (2) Houses without any window spaces incorporated into their design, so their eaves could not be closed without compromising ventilation and lighting.

For the first group of houses, the pre-existing practice of filling window spaces with brick lattice work (Fig. 7) was identified as a widely acceptable medium-term substitute for security bars, which allowed more airflow and light to enter than the previously near-complete solid temporary brickwork filling (Fig. 4A,B,C,D). While this practice was far from universal in the community at the outset of the study, this solution proved acceptable to almost all (85%; 35/41) participating households living in this first category of houses with window spaces incorporated into their design.

Wherever the household head agreed, the research team took the opportunity to open up window spaces as much as possible by reorganizing the pre-existing brick work filling into lattices, thus allowing more airflow and light into the house (Fig. 7A, B), especially in houses with closed eaves and limited ventilation generally.

**Fig. 8:**
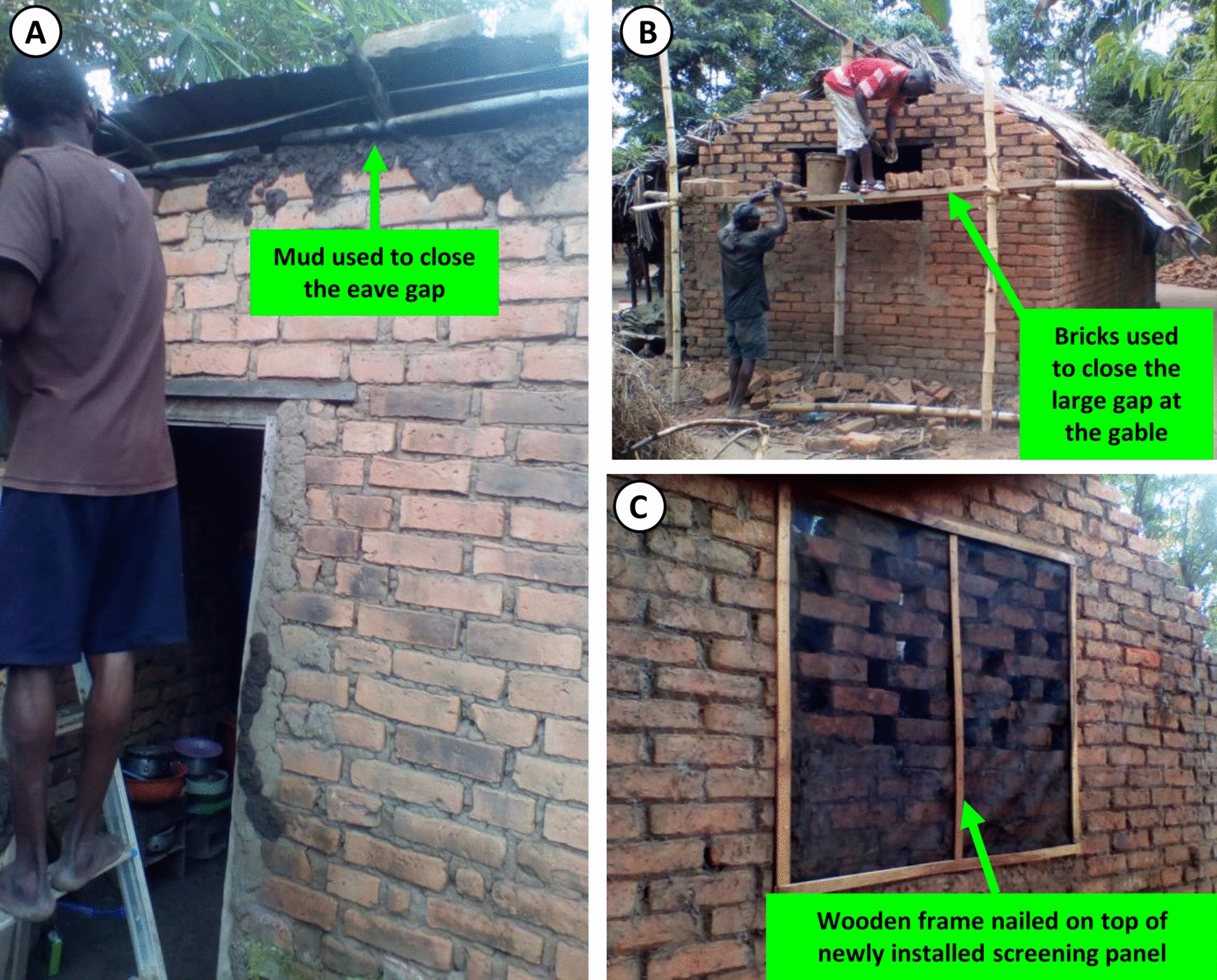
Examples of occupant investments from their own pockets into enhancing the housing modifications they were helped to implement by the research team. **A** Illustrates one household who enthusiastically closed the eaves with mud and bricks. **B** A household that bought their own bricks and used them to fill in their very large eave gaps to facilitate screening of the remaining openings by the research team. **C** An example of how one household bought and installed wooden strips to re-enforce the netting screen installations which were provided free of charge by the study team

**Fig. 9:**
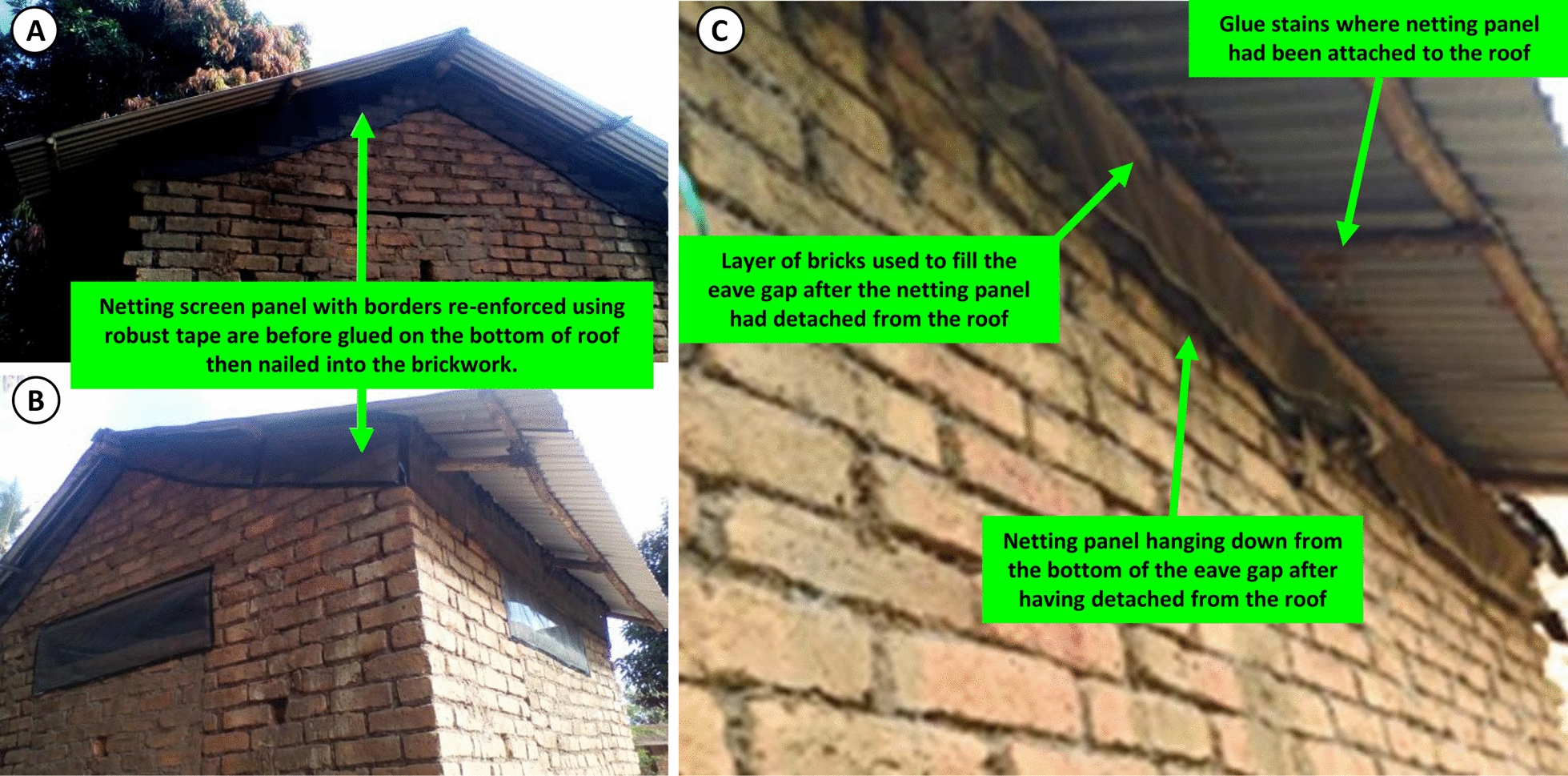
The panels show how some of the larger  brick houses with metal roofs fitted with netting on their eaves following the request of house owners. **A**, **B** Are both representative the kind of large houses that required a lot of netting, tape, nails, glue and other sundry materials to screen their eaves. The netting screens were first glued to bottom of the iron roof and then other hanging part was nailed on to the walls. Unfortunately, this particular installation format never lasted more than a year after installation (**C**), due to detachment of the glued section from the iron roof sheets. As illustrated in **A**, in all cases brick houses requesting netting eave screens declined to have the brickwork in their window spaces reorganized into lattices

Many householders expressed clear awareness of the health benefits of increased air and light within their houses but also substantive concerns about risks to their privacy and security, particularly their vulnerability to thieves. Despite these concerns, 94% (31/33) of households with open eaves agreed to close them with readily available brick rubble and mud (Fig. 8A) once their window spaces had been modified into open brickwork lattices (Fig. 7) and 3 households even purchased concrete (Fig. 8B) and wooden strips (Fig. 8C) to enhance these modifications at their own expense. Initially, a small number of households (4% or 2/41 of the well-ventilated houses) preferred fitting tape-reinforced eave screening panels over closing their eaves (Fig. 9A, B). In both cases, the householders explained that this preference was motivated by the need to prevent ants or termites from attacking the wooden beams or branches supporting the roof. However, installing eave screening to these houses proved very awkward and laborious to implement because these panels had to be individually tailored to fit around the numerous wooden beams supporting the roof sheeting and could only be secured along their upper edge by gluing them to the undersides of the metal sheets of the roof (Fig. 9C). Also, the latter necessity yielded an installation format that proved to have poor durability, so some of these households (33%; 2/6) subsequently decided to close their eaves with brick and mud (Fig. 8), similarly to those households who agreed to this modification from the outset.

In summary, such large brick houses in the first category of structural modification needs, with bricked up window spaces and open eaves, were far more laborious and expensive to screen because these eave gaps are not only physically awkward but also extensive in length (Table 1, Figs. 2 and 3), so they required far more netting and additional materials per unit of footprint area to screen effectively (Fig. 9).

**Fig. 10:**
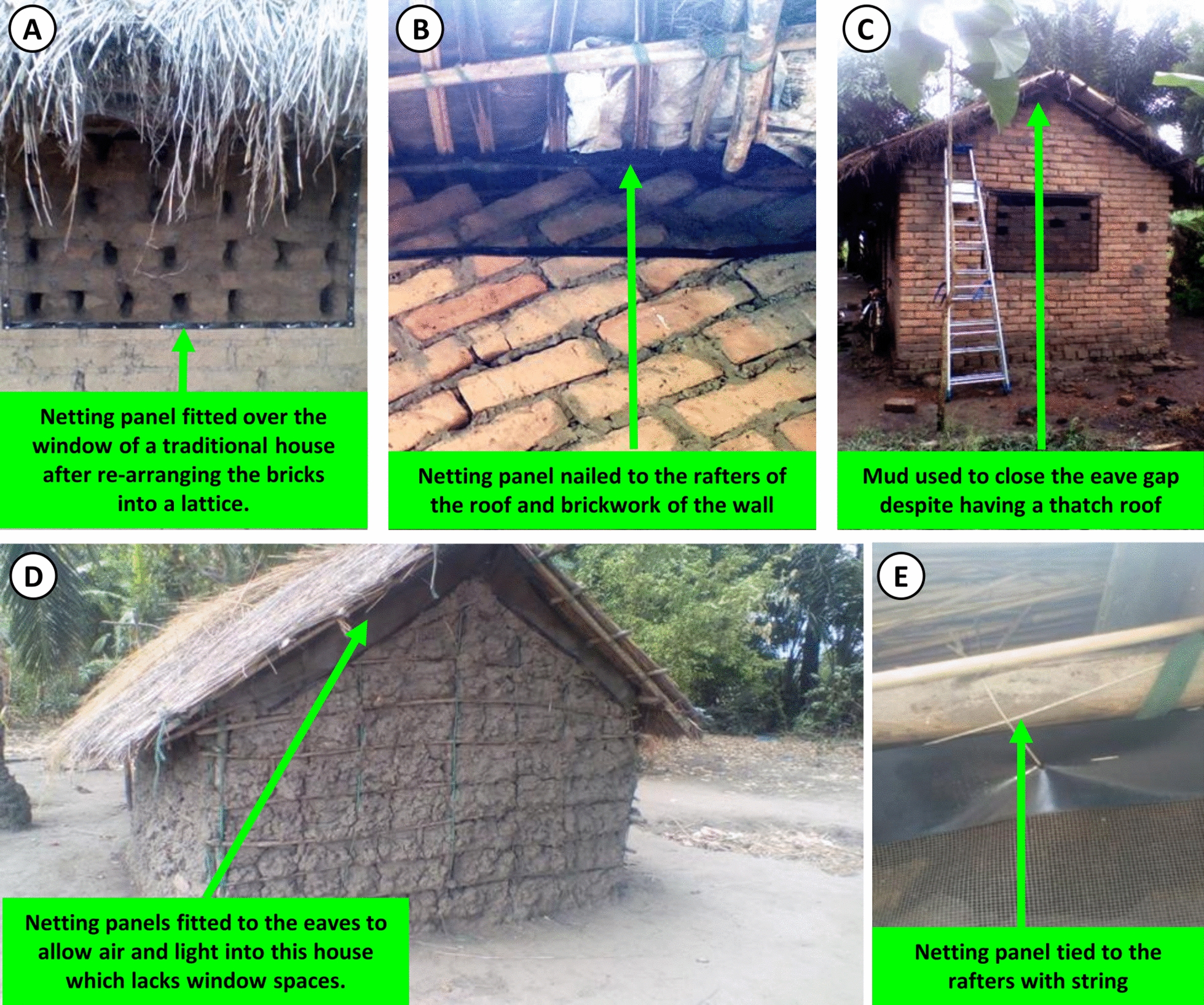
Examples of netting screen installations in houses built with traditional materials. **A** A typical traditional house made with unbaked bricks showing how these houses can be modified in order to allow air and light to pass into the house through brick lattices that can then be screened to exclude mosquitoes as described in Fig. 6 for more contemporary house designs. **B**–**D** depict houses with thatched roofs over baked brick (**B**, **C**) or mud and wattle (**D**) walls. While **C** illustrates one example in which the household chose to block the eaves with brick rubble and mud, the majority of households with thatched roofs preferred installation of netting eave screens (**B**, **D**), to prevent ants and termites attacking the natural materials they are constructed from. **E** illustrates how easily the netting screen could be installed over the eaves of thatched roofs by nailing or tying directly onto the bamboo or palm stem supports holding up the roof

For the second group of houses lacking window spaces, either the eaves were screened with netting panels (Fig. 10B) or they were filled with brick rubble and mud (Fig. 10C), depending on the preferences of the households themselves, exactly as described for the houses with window spaces. On the other hand, none of the mud and wattle houses in this poorly ventilated housing cluster required any window screen installations, simply because they all lacked windows and relied entirely upon open eave gaps for ventilation (Fig. 10D). Fortunately, the eave gaps of these windowless mud, wattle and thatch houses, or even brick-walled houses with thatched roofs proved to be far easier and less expensive to screen with netting than larger brick houses with metal roofs. This was simply because screen panels with re-enforced borders could be readily tied/nailed to the bamboo/palm stem lattice supporting the thatch (Fig. 10E).

It was also observed that occupants of mud and wattle houses often left gaps in the walls or around the door frames and refrained from filling them because there was little point in doing so when the eaves remain open, because mosquitoes would consequently find their way in regardless. Following screening of the open gaps of eaves in two of the five mud and wattle houses, the investigators succeeded in convincing the occupants to fill such gaps in the walls and around door frames with mud after explaining to them that these had probably become the most important remaining entry point for malaria mosquitoes.

One particularly useful experience to communicate from the extensive informal discussions with householders was the importance of explaining the known differential effect of house screening upon house entry by *Anopheles* and *Culex* mosquitoes [45, 46]. Almost all households were unaware that most of the mosquitoes in their houses were *Culex* mosquitoes and that they are incapable of transmitting malaria but readily find their way into even the best-screened houses [47,48] for reasons that remain to be determined. Once this difference between *Culex* and *Anopheles,* in terms of their ability to enter screened houses, and the known potential for selectively excluding malaria vectors even from houses with persisting high densities of *Culex* was explained, all participants then engaged far more enthusiastically in the study.

### Effect of screening eaves and windows on indoor densities of mosquitoes

A total of 110,984 female mosquitoes were collected over a total of 921 house nights of trapping over the full course of the experiment, averaging 121 mosquitoes per house per night. The most abundant mosquito taxon caught was *Culex spp.* (107,678 or 97.0% of the total), most of which were probably *Cx. quinquefasciatus* [49]. Most of the small remainder (2543 or 2.3%) were identified morphologically as members of the *An. gambiae* complex, which was entirely composed of *An. arabiensis* in the area at the time of the study [31]. *Mansonia africana* and *Anopheles squamosus* respectively represented about 0.5% (549) and 0.2% (198) of the total mosquito catch, but neither are considered to be vectors of any common disease in this area. The least abundant mosquito taxon caught was the *An. funestus* group, of which only 16 specimens were caught, accounting for only 0.1% of the total catch. The statistical analysis of indoor mosquito densities therefore focused upon *Culex* spp. as the most important cause of nuisance biting and *An. gambiae *sensu lato (*s.l*.) as the most important malaria vector in this study setting at that time. From this point forward, counts for these taxa are described as *Cx. quinquefasciatus* and *An. arabiensis* on the basis of the respective dominance of these two species within these two taxa [31, 49].

**Fig. 11:**
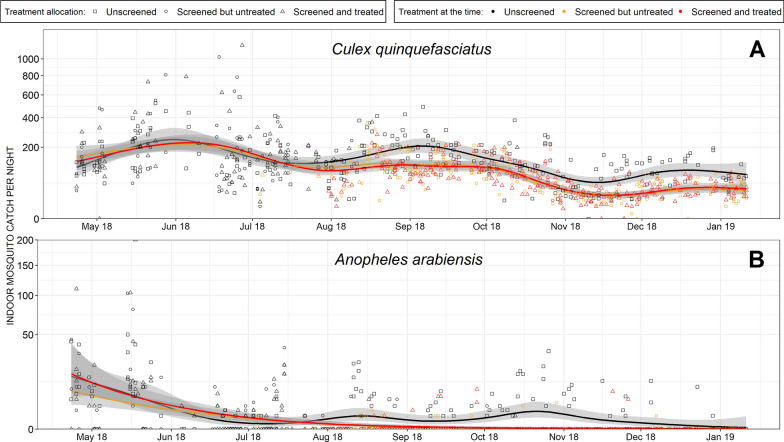
Indoor catches of *Cx. quinquefasciatus* (**A**) and *An. arabiensis* (**B**) with the smoothed mean for each treatment allocation arm estimated with a generalized linear mixed model (GLMM) for each species outlined as the black (Negative control; no screening), yellow (Screening but no insecticide treatment) and red (Insecticide-treated screens) lines with their respective 95% confidence intervals depicted as grey shading. The dependent variable was density of indoor collected mosquitoes, while screening procedure was the independent variable and the random variables were the time (date when data were collected) and location (the neighbourhood each houses was recruited from)

As illustrated in Fig. 11A, houses in all three of the allocated study arms had similar indoor densities of *Cx. quinquefasciatus* before the introduction of screening as an intervention (P = 0.65 and P = 0.91 for houses randomly allocated to screened but untreated or screened and those screens treated with PM, respectively, compared to houses allocated to unscreened arm). Similarly, Fig. 11B illustrates how indoor densities of *An. arabiensis* were indistinguishable across the three study arms before any screening intervention treatments were introduced (P = 0.13 and P = 0.93 for houses respectively allocated to screened but untreated and those assigned to screened plus PM treatment, when compared to houses allocated to the unscreened study arm). Once screening was introduced, however, indoor catches of both species were reduced in houses assigned to those two study arms (Fig. 11A, B and Table 2). Consistent with previous reports [47, 48, 50], the degree of reduction observed differed starkly between the two mosquito species: While indoor densities of *Cx. quinquefasciatus* were approximately halved, *An. arabiensis* densities were reduced by approximately 40-fold, relative to houses in the controls. Treatment of the netting screens with PM insecticide had no apparent effect on indoor catches of *Cx. quinquefasciatus* or *An. arabiensis* (P = 0.831 and 0.676, respectively for houses with insecticide-treated screens versus those with untreated screens).

**Table 2:** Impact of screening with and without insecticide treatment upon the densities of *Culex quinquefasciatus* and *Anopheles arabiensis* as estimated with a separate generalized linear mixed model for each species

Mosquito taxon and treatment arm allocation	Post-intervention mosquito density expressed as absolute rates [95% CI] (Mosquitoes·house^−1^ ·night^−1^)	Estimated intervention effects expressed as relative rates [95% CI]	P
*Culex quinquefasciatus*			
No screens	76 [66, 87]	1.00	NA
Screened but untreated	40 [35, 47]	0.53 [0.46, 0.62]	<< 0.0001
Screened and treated	41 [35, 48]	0.54 [0.46, 0.63]	<< 0.0001
*Anopheles arabiensis*			
No screens	0.22 [0.13, 0.39]	1.00	NA
Screened but untreated	0.007 [0.003, 0.016]	0.029 [0.013, 0.065]	<< 0.0001
Screened and treated	0.005 [0.002, 0.014]	0.023 [0.010, 0.055]	<< 0.0001

Here the dependent variables were the numbers of mosquitoes collected indoors, screening status (treated and untreated screens pooled into a single category versus unscreened as the reference category) was the independent variable of interest

While well-ventilated houses had similar indoor mosquito densities to poorly ventilated ones in the absences of house screening, there was an interactive effect between the two factors that approached significance for *Cx. quinquefasciatus* and was highly significant for *An. arabiensis* (Table 3). Once this incremental combination effect was accounted for, poorly ventilated houses were found to experience a somewhat lower level of protection against indoor-biting *An. arabiensis* (88% in Table 3 versus 97% in Table 2). However, well ventilated houses were found to accrue a further 89% incremental reduction of *An. arabiensis* densities when screened, resulting in an overall impact of 98%. While a similar interaction effect between screening and ventilation planning approached statistical significance for *Cx. quinquefasciatus* (Table 3), the small size of that incremental effect (20% reduction) renders it of negligible relevance to pathogen transmission or the perceptions of householders regarding the effectiveness of screening for preventing biting nuisance.

**Table 3:** The simple direct effects of screening (treated and untreated screens pooled into a single category based on similarity (Table 2) versus unscreened houses as the reference group) and house structure cluster membership (well ventilated versus poorly ventilated as the reference group) upon indoor mosquito densities, as well as the interactive effect between these two factors (screened and well ventilated versus all other combinations combined as the reference group), as estimated with a single generalized linear mixed model for each species

Attribute	Relative rate [95% CI]	p-value
*Culex quinquefasciatus*		
Screened	0.56 [0.44–0.70]	< 0.0001
Well ventilated	1.07 [0.87–1.31]	0.51
Screened × well-ventilated interaction	0.80 [0.62–1.03]	0.08
*An. Arabiensis*		
Screened	0.12 [0.04–0.31]	< 0.0001
Well ventilated	1.43 [0.68–3.00]	0.35
Screened × well-ventilated interaction	0.11 [0.03–0.40]	0.0008

### Residual insecticidal efficacy of pirimiphos-methyl painted onto mosquito screens

**Fig. 12:**
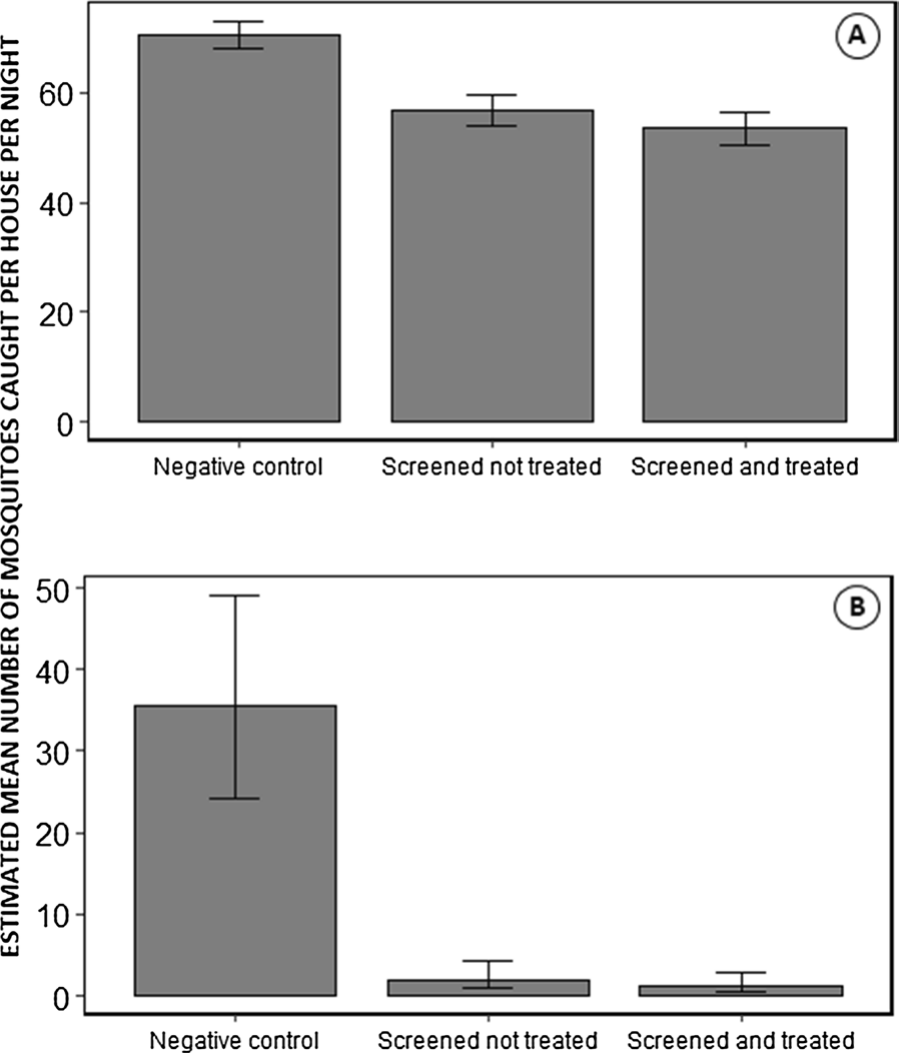
Mean densities of mosquitoes caught in houses following implementation of the intervention treatments assigned to each house in the study design (Fig. 1)

**Fig. 13:**
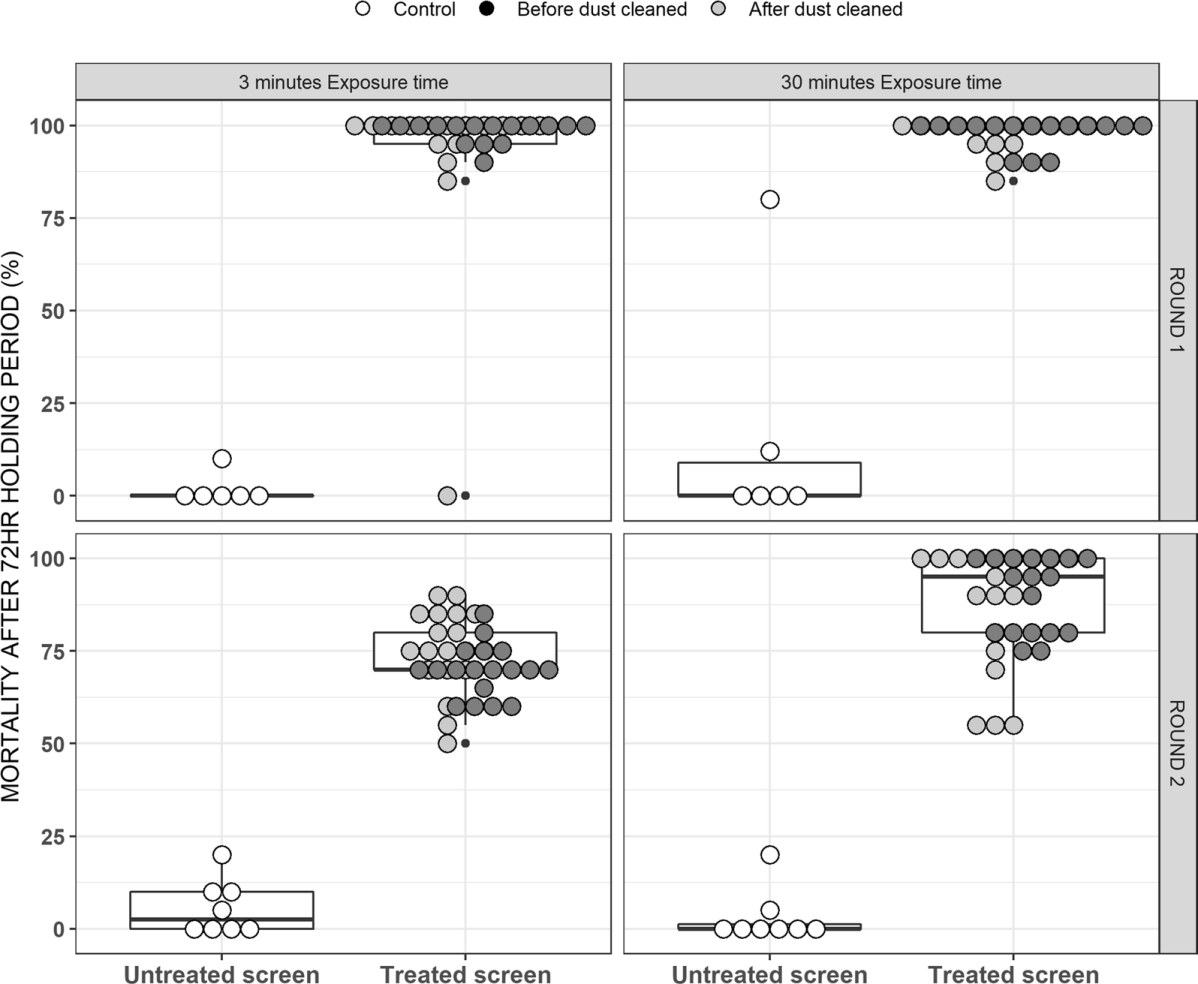
The results of standard WHO cone assay tests for the residual efficacy of the pirimiphos-methyl (PM) treatments of mosquito screens fitted to participating houses. These assays used insectary-reared *An. gambiae* Ifakara strain and were conducted for 9 houses that were screened but not treated and for 17 houses that were screened and then the screens were then treated with PM. For each house on each round of data collection, only one screen installed over either an eave gap or a window was picked arbitrarily and tested at 4 different arbitrarily selected points

The PM treatments of the netting screen installations retained very high levels of residual efficacy against susceptible insectary-reared mosquitoes 4 months after initial treatment and reduced but nevertheless satisfactory efficacy after 8 months (Fig. 12). Wiping dust off the screens had no effect upon the insecticidal efficacy of the treatments (P = 0.42). Overall, insecticide activity was slightly reduced 8 months after PM treatment (Proportional mortality [95% CI] = 89% [83%, 93%]) when compared with the survey 4 months post treatment (Proportional mortality [95% CI] = 99% [97%, 99%]) when mosquitoes were exposed to the screens for 30 min and then maintained under insectary condition for 72 h (Odds ratio [95% CI] = 0.1 [0.06, 0.17], p < 0.0001) (Fig. 13).

## Discussion

Overall, the participatory community engagement approach of this study proved effective, with only two enrolled houses withdrawing from the study. All the remaining 52 houses participated enthusiastically throughout and were successfully modified and screened during or at the end of the study. Furthermore, collaboration with the participating households allowed us to identify common, freely available materials in the environment immediately surrounding each house and exploit them for the purpose of modifying their structure to make them easier and more affordable to screen. While some households had clear preferences about which structural modifications they found acceptable, all of them agreed to make necessary modifications and then agreed to the installation of netting screens to protect themselves from being bitten by mosquitoes while indoors. These simple and practical house modifications were made almost exclusively with locally available materials and were clearly understood by participants as a temporary substitute for far more expensive conventional window screening installations with permanent wooden frames and metal security bars and/or grills. These findings are consistent with those reported for similar participatory approach in Mozambique where households also engaged enthusiastically and some similar technical solutions were developed, specifically the fitting of cloth borders to screen netting panels [51]. The success of the participatory approach applied and the technical solutions developed through this small pilot study in Tanzania, and previously in Mozambique [51], suggest that the usual obstacles to mosquito proofing faced by low income households with so many other competing budget priorities [11, 37, 52, 53] may well be possible to overcome. Further studies that extend such approaches across much larger populations, and which include formal costing and social science investigations, are clearly merited to more rigorously assess their scalability.

Consistent with dozens of previous reports, this study confirms that screening of houses reduces indoor mosquito densities, even without any insecticide [8, 9, 54]. Furthermore, the observations confirm that house screening has a far greater impact upon indoor densities of *Anopheles*, in this case *An. arabiensis* (98% reduction), than upon the *Cx. quinquefasciatus* (46% reduction) [47, 48, 50]. The ability of *Cx. quinquefasciatus* to maintain high levels of nuisance biting inside screened houses, despite almost complete exclusion of the *Anopheles* species that mediate malaria transmission, was consistent with the results of previous studies [50]. This sustained biting nuisance by *Cx. quinquefasciatus* specifically was observed to undermine community perceptions of efficacy and acceptability until discussed with them and explained the distinct morphology and roles of these two mosquito taxa. Given that *Cx. quinquefasciatus*, otherwise known as the Southern House Mosquito, is probably the world’s most abundant human-biting mosquito [55, 56],﻿ and is known to motivate uptake of vector control measures like repellent coils [57], its influence upon public enthusiasm for screening of houses merits careful consideration and detailed investigation.

Unfortunately, the PM insecticide treatment evaluated here did not augment the household-level of protection provided by the physical barrier of the netting screens against either mosquito species. More encouragingly, however, this insecticide treatment did exhibit levels of residual efficacy against insectary-reared *An. gambiae* that remained very high 4 months after initial treatment and robustly satisfactory after 8 months, despite being exposed to dust and sunlight outdoors. So, while treating the screens in this way added no direct incremental protection at household level, it may nevertheless be possible to achieve more important community level effects upon malaria transmission through mass vector population suppression if high enough coverage can be achieved across landscape scales [16, 23–26].

One interesting and unforeseen observation of this study was the dichotomy of house designs that separated into distinct clusters of designs with well-planned and poorly planned structures in terms of potential for ventilation. Even more interesting was the interaction between the two design categories and the household-level protective effects of mosquito proof screening, with well-ventilated housing designs responding appreciably better to screening in terms of relative reductions of both *An. arabiensis* and *Cx quinquefasciatus* densities. Although this difference between the two distinct clusters of housing designs could have arisen from covariance between the design and the materials these houses were built with, this seems unlikely given that all these variables were included in the cluster analysis and a recent experimental study confirms that good ventilation can reduce mosquito entry rates [58].

While these results are generally very encouraging, it must also be recognized that this study has several limitations that merit detailed consideration. Indeed, all these study limitations will need to be addressed by further research studies before the effectiveness of this approach at scale can be adequately assessed and the generalizability of the findings across settings determined.

First and foremost, this was ultimately a relatively small study, with only 52 houses from the fringes of one modestly sized town in southern Tanzania enrolled and retained throughout the duration of the study. The representativeness of this small sample of houses and the generalizability of the results are therefore open to question. Second, the highly selective inclusion criteria used to enrol only houses with some remaining open entry points for mosquitoes was rational but obviously constrains extrapolations beyond these direct observations to expectations under conditions of community-wide scale up. Fully completed houses which had already been well screened were deliberately excluded, allowing us to focus on the kinds of houses that really needed interim mosquito proofing solutions while their occupants saved up for the expensive window frames and security bars required to install window screens on an essentially permanent basis. This study included no formal or representative community-wide surveys of the distributions of housing structures, ventilation planning or degree of mosquito-proofing across the community at large, so it is not possible to be sure how relevant the results obtained from this small and intentionally biased sample of houses are to the overall housing quality picture in Ifakara town, much less elsewhere in Tanzania or other African countries. Further studies should, therefore, be larger in scale and should attempt to estimate the size of the coverage gap this participatory approach could help fill, as well as the degree of coverage achieved in practice across entire communities.

Future studies should also ideally include far more formal social science investigations that go into far greater depth and triangulate the results of complementary methodologies against each other, such as in-depth interviews, focus group discussions and *Photovoice* surveys [14]. While some limited formal social science assessments had been planned to document the perspectives and ideas of the participant households at the end of this study, that was not possible for the same political and financial reasons described below in relation to the limited period of follow up on the insecticidal efficacy of the treated screens.

While only entomological indicators of protection against exposure to malaria vectors were recorded as the primary outcome, that makes sense in the context of such a small exploratory pilot study. Indeed, the limited scale of this study, and randomization at the level of individual houses rather than entire village communities, both precluded achievement of community-level mass effects on mosquito populations. It would, therefore, have been premature to record epidemiological indicators, which would in any case have been grossly underpowered for the purposes of demonstrating actual impacts upon malaria infection prevalence or incidence [59]. Consistent with current recommendations [59, 60], it was therefore considered reasonable to focus on entomological indicators of the direct personal protection effects that could be measured with such randomization at house level, as well as the insecticidal efficacy of the treated screens as an indicator of potential for community-wide mass effects on mosquito populations if implemented across larger scales.

However, perhaps the greatest limitation of this study is that measurements of insecticidal activity on the treated netting screens were only conducted twice at 4 months and 8 months, without further follow up. While the levels of insecticidal activity observed were encouragingly high at both time points, even without cleaning dust from the netting, it is only possible to speculate how long it would have lasted beyond that or how often such screens would need to be retreated. This relatively short period of follow up was caused first by an unfortunate turn of political events, when a round of local elections resulted in the majority of the local leaders who had facilitated the study being replaced, following which the term of the funding award that supported it expired before it was possible to re-establish collaboration with these new chairpersons and TCU leaders.

## Conclusion

Despite these limitations, this small-scale pilot study does establish encouraging proof of principle for a number of intervention opportunities worthy of further investigation. First of all, community members proved largely receptive to the participatory approach applied here, resulting in comprehensive success with screening houses to effectively exclude malaria vectors, in most cases after making enabling structural modifications that were widely accepted by the enrolled households. While the insecticide treatment applied to these netting screens conferred no apparent incremental protection against mosquito entry at household level, the durability of insecticidal activity observed for this IRS formulation on the netting screens was encouraging: The observation that PM insecticide activity was sustained for at least 8 months on treated netting screens, which had far smaller combined surface areas than the walls and ceilings that this IRS formulation would normally be applied to [15], suggests that sizeable community-level mass effects upon malaria vector populations may be reasonable expected if this intervention were rolled out across larger scales. It is also encouraging that these screen installations proved considerably more effective at excluding malaria vector mosquitoes from more contemporary house designs intended to enable improved ventilation, so it may well be possible to achieve synergy between this supplementary housing improvement intervention and existing efforts of households to improve their domestic living environments.

## Supplementary Information

**Additional file 1.** An Excel® spreadsheet file containing all three data tables that were collected and analysed in this study, anonymized by removing all variables containing information that could be used to identify individuals, households or their houses.

## Data Availability

All of the materials used are freely available on the open market, including the formulation of PM insecticide used, which is registered in dozens of countries across Africa. The datasets and images supporting the conclusions of this article are included within the article and Additional file 1.

## Abbreviations

IRS: Indoor residual spraying
ITNs: Insecticide-treated nets
PM: Pirimiphos-methyl
WHO: World Health Organization
